# Updated Evaluation of Laparoscopic vs. Open Appendicectomy During Pregnancy: A Systematic Review and Meta-Analysis

**DOI:** 10.3389/fsurg.2021.720351

**Published:** 2021-09-23

**Authors:** Jia Zhang, Miye Wang, Zechang Xin, Ping Li, Qingbo Feng

**Affiliations:** ^1^Department of Breast Surgery, West China Hospital of Sichuan University, Chengdu, China; ^2^Engineering Research Center of Medical Information Technology, Ministry of Education, West China Hospital, Sichuan University, Chengdu, China; ^3^Information Technology Center, West China Hospital of Sichuan University, Chengdu, China; ^4^Key Laboratory of Carcinogenesis and Translational Research (Ministry of Education/Beijing), Department of Hepatobiliary and Pancreatic Surgery Unit I, Peking University Cancer Hospital and Institute, Beijing, China; ^5^Northern Jiangsu People's Hospital, Clinic Medical College, Yangzhou University, Yangzhou, China; ^6^Department of Liver Surgery and Liver Transplantation Centre, State Key Laboratory of Biotherapy and Cancer Center, West China Hospital, Sichuan University, Chengdu, China

**Keywords:** appendicitis, laparoscopic appendectomy, open appendectomy, pregnant, fetal loss

## Abstract

To explore the updated evaluation about the obstetrical and perioperative outcomes of laparoscopic appendicectomy (LA) for pregnancy appendicitis compared with open appendicectomy (OA). Two reviewers independently searched the PubMed, the Cochrane Central Register of Controlled Trials, EMBASE, and Web of Science databases to screen eligible studies up to December 2020. Only clinical researches, no < 10 cases for LA and OA group were included. Twenty retrospective studies with 7,248 pregnant women, evaluating LA and OA in surgical and obstetrical outcomes, were included. The weighted mean difference (WMD) with 95% CI and odds ratio (OR) was used to compare continuous and dichotomous variables. It seems LA was connected with significantly shorter hospital time and lower wound infection [mean difference (MD), −0.57 days; 95% CI, −0.96 to −0.18; *p* = 0.004 and OR, 0.34; 95% CI, 0.18 to 0.62; *p* = 0.0005, respectively]. The incidence of fetal loss after LA was higher than OA (OR,1.93; 95% CI, 1.39–2.69; *p* < 0.0001). It was almost similar in the rate of preterm delivery (OR, 0.80; 95% CI, 0.48 to 1.34; *p* = 0.40) and other perioperative and obstetrical complications (*p* > 0.05). Our results indicated that the occurrence of fetal loss after LA should not be ignored. Caution, skillful operation, and thoroughly informed consent about the advantages and disadvantages of laparoscopy are necessary.

**Systematic Review Registration:** https://www.crd.york.ac.uk/PROSPERO/#recordDetails, identifier: CRD42021233150.

## Introduction

Acute appendicitis, the most common cause of non-obstetric acute abdomen, affecting 1.8–41 per 10,000 pregnancies and one in nearly 1,000 births (1, 2), accounts for 25% of non-obstetric procedures performed in the prenatal period (3). Acute appendicitis during pregnancy possesses a unique treatment challenge because of the high probability of Appendix translocation and the presence of the fetus limiting the applicability of imaging techniques (4). In addition, the symptoms and signs of appendicitis usually include lower abdominal pain, nausea, and lack of appetite, occasionally similar to the onset of pregnancy or parturition (5). Delayed diagnosis and treatment both increase the threats of perforation and intraabdominal infection that is associated with growing motherly morbidity and fetal mortality (6). For suspected pregnant appendicitis, open or laparoscopic appendectomy (LA) is the mainstay of treatment strategy (7, 8).

According to previous reports, patients with LA had less wound infection, less post-operative ache, and get back faster to work than those with open appendectomy (OA) (9, 10). Laparoscopies give a clearer visualization of the whole abdomen than OA. The Society of American Gastrointestinal and Endoscopic Surgeons (SAGES) guidelines currently is inclined to LA as the routine therapy for pregnant appendicitis (11). However, some large-scale studies have pointed out that LA has a non-ignorable influence on fetal loss and pre-term labor (12, 13). The optimal surgical procedure is still equivocal for pregnancy appendicitis. Acute simple appendicitis tends to occur in the first and second trimesters, whereas perforation is more inclined toward the third trimester (5, 6, 14). However, in clinical practice, LA and OA both have been performed in patients, whether it is the early or the third trimester. Many systematic reviews have been published to evaluate the effects of the two surgical methods on the fetus and pregnant women, but until now there have been no consistent conclusions (15–17). In the past 2 years, some clinical studies evaluating the two operations have tended to show no significant difference in obstetric outcomes, such as fetal loss and pre-term delivery (18, 19). It is likely that the obstetric outcomes may have improved with recent progress in perioperative care and laparoscopic technique. To illustrate these issues, we implemented a systematic review and updated meta-analysis to evaluate the obstetric and operational outcomes of LA and OA during pregnancy.

## Methods

### Materials and Methods

The study has been conducted on the basis of the PRISMA guidelines (20) and registered with a registration number at PROSPERO (CRD42021233150). Institutional Review Board approval is not required as this article is a systematic review and not an experimental study.

### Search Strategy

A literature retrieval was conducted on December 23, 2020. Major electronic literature databases, PubMed, Web of Science, EMBASE, and the Cochrane Central Register of Controlled Trials, were roundly screened by two independent investigators (J Zhang and MY Wang) using the following search terms: pregnancy, laparoscopic, open, appendicectomy and appendicitis. The particular search strategies are in Appendix. To prevent missing relevant studies, the references of literature and publications were manually searched. We used the endnote X9 to remove the duplicated articles.

### Inclusion and Exclusion

Two investigators (J Zhang and MY Wang) reviewed the currently available literature and independently viewed all titles and abstracts for eligible studies. Included in the standard were the following: (I) research on LA and OA for pregnancy appendicitis; (II) randomized controlled trials (RCTs), case-control studies, retrospective studies, and cohort studies; (III) pregnant patients with acute appendicitis. Exclusion criteria included the following: (I) editorials, reviews, letters, comments, animal studies, or case reports; (II) non-English studies; (III) studies < 10 patients per group; (IV) data that were unrecognizable and incomplete.

### Data Extraction and Evaluation

Data extraction included the following items: study and patient traits, which included the year of publication, first author, type of study, sample sizes, average age, research time, gestational age at surgery, conversion to OA, country, trimesters, pathology of appendicitis; operative and obstetrical outcomes, which included fetal loss, pre-term delivery, cesarean section, surgery time, length of stay (LOS), wound infection, abscess, and post-operative uterine contraction. The primary outcomes were fetal loss and pre-term delivery, the secondary outcomes included cesarean section, LOS, operative time, wound infection, abscess, and post-operative uterine contraction. Two reviewers (J Zhang and MY Wang) independently extracted the information using a uniform format, and if there was any ambiguity, a third observer (QB Feng) joined to reach a consensus. We utilized the Newcastle–Ottawa Scale (NOS) to comprehensively evaluate included studies as all eligible studies were retrospective (21). Every study was independently evaluated by two reviewers (J Zhang and MY Wang), and a NOS score of ≥ 6 is considered a sign of high quality.

### Statistical Analysis

We used Review Manager 5.3 Software to analyze the data. The weighted mean difference (WMD) with the 95% CI and odds ratio (OR) was used to compare continuous and dichotomous variables. Studies reporting median results for continuous data were converted to mean and SD if the median range and sample volume were recorded (22). The diverse heterogeneity of the included studies was evaluated by the *I*^2^-test. If *I*^2^ < 50%, the heterogeneity was considered slight, and the fixed-effect model was wielded to perform pooled OR and WMD (23); if not, the random-effects model was used (24). The funnel plot was adopted to assess the publication bias (25). For excluding the potential influence of individual studies on the main results, we step by step exclude individual studies to analyze their impact on the overall results.

## Results

### Retrieved Results and Features of the Appropriate Studies

**Figure 1:**
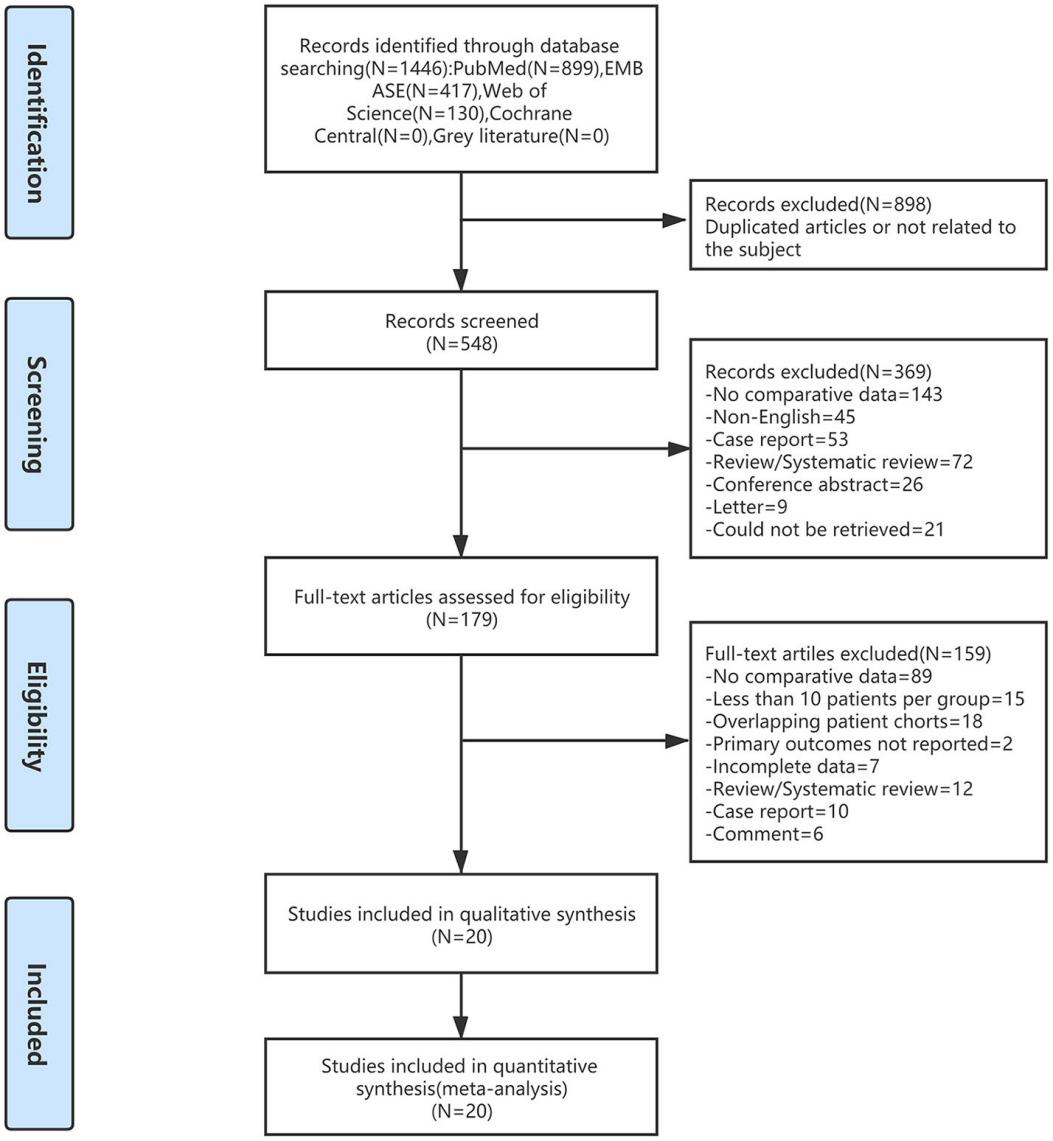
Flow diagram of study identification and selection. LA, laparoscopic appendectomy; OA, open appendectomy.

The literature retrieval outputted 1,466 correlative English publications from the multifarious electronic databases, of which 20 retrospective studies (12, 13, 18, 19, 26–41) comparing in a total of 7,248 pregnant women (2,477 and 4,771 patients underwent LA and OA, severally) were screened for farther analysis. The flow diagram of our analysis schedule is presented in Figure 1. The primary characteristics of the included 20 studies are shown in Table 1, whereas the surgical and obstetrical results of the included papers are listed in Table 2. All results of our systematic review are presented in Table 3. Meanwhile, the quality evaluation of included studies with the Newcastle Ottawa Scale is presented as a summary in Table 4.

**Table 1:** Study characteristics and qualities.

**References**	**Type of study**	**Patients (LAvsOA)**	**Age (years)**	**Research time**	Center^*^	Transition^*^	**Country**	**Trimesters of pregnancy**			**NOS**
Winter et al. (12)	Retrospective	125 vs. 93	27 ± 8 vs. 28 ± 8	2000–2012	Multicenter	1	Australia	1st	2nd	3rd	8
Segev et al. (40)	Retrospective	50 vs. 42	28 ± 9 vs. 29 ± 7	2000–2014	Monocenter	1	Israel	1st	2nd	3rd	8
Laustsen et al. (36)	Retrospective	19 vs. 25	30.5 vs. 30.5	2000–2012	Monocenter	1	Denmark	1st	2nd	3rd	7
McGory et al. (13)	Retrospective	454 vs. 2,679	N/A	1995–2002	Multicenter	0	USA	1st	2nd	3rd	8
Affleck et al. (27)	Retrospective	22 vs. 18	N/A	1990–1998	Monocenter	1	USA	1st	2nd	3rd	6
Carver et al. (29)	Retrospective	17 vs. 11	23 ± 5 vs. 24 ± 7	2000–2002	Monocenter	0	USA	1st	2nd	None	7
Karaman et al. (34)	Retrospective	12 vs. 36	27.08 ± 5.48 vs. 28.81 ± 8.35	2010–2015	Multicenter	0	Turkey	1st	2nd	3rd	8
Cheng et al. (30)	Retrospective	128 vs. 653	N/A	2005–2010	Multicenter	0	China	N/A	N/A	N/A	7
Peled et al. (38)	Retrospective	26 vs. 59	29.2 ± 4.9 vs. 27.6 ± 4.7	2000–2009	Monocenter	0	Israel	N/A	2nd	N/A	7
Chung et al. (31)	Retrospective	22 vs. 39	29.3 ± 3.1 vs. 31.4 ± 4.3	2007–2011	Monocenter	0	Korea	1st	2nd	3rd	7
Eom et al. (33)	Retrospective	15 vs. 28	27.2 ± 4.04 vs. 30 ± 3.75	2000–2010	Monocenter	0	Korea	1st	2nd	3rd	7
Sadot et al. (39)	Retrospective	48 vs. 17	29.79 ± 6.2 vs. 28.76 ± 5.1	1999–2008	Multicenter	0	USA	1st	2nd	3rd	7
Cox et al. (32)	Retrospective	894 vs. 441	27.7 ± 6.2 vs. 28.2 ± 6.3	2005–2012	Multicenter	0	USA	N/A	N/A	N/A	7
Gok et al. (18)	Retrospective	18 vs. 39	30 ± 5 vs. 27 ± 5.5	2009–2018	Monocenter	0	Turkey	1st	2nd	3rd	7
Kirshtein et al. (35)	Retrospective	23 vs. 19	29.8 vs. 26.8	1997–2007	Monocenter	1	Israel	1st	2nd	None	6
Lyass et al. (37)	Retrospective	11 vs. 11	28.5 ± 5.27 vs. 30 ± 6.93	1996–1999	Monocenter	0	Israel	1st	2nd	3rd	8
Yoo et al. (41)	Retrospective	24 vs. 56	30.2 ± 2.9 vs. 31 ± 4.8	2008–2015	Multicenter	0	Korea	1st	2nd	3rd	7
Tumati et al. (19)	Retrospective	547 vs. 459	27 ± 9 vs. 27 ± 10	2005–2014	Multicenter	0	USA	1st	2nd	3rd	8
Cai et al. (28)	Retrospective	12 vs. 36	27.5 ± 5.5 vs. 28.3 ± 5.6	2016–2018	Monocenter	0	China	None	2nd	None	8
Kapan et al. (26)	Retrospective	10 vs. 10	27.1 ± 4.28 vs. 25 ± 4.5	2009–2011	Monocenter	0	Turkey	N/A	N/A	N/A	7

Center^*^: the number of center; monocenter =1, multicenter ≥ 2. Transition^*^: 1 = Yes, 0 = No.

*LA, laparoscopic appendectomy; OA, open appendectomy; N/A, not available.*

**Table 2:** Obstetrical and surgical outcomes of included studies (LA vs. OA).

**References**	**Length of stay (days)**	**Wound infection**	**Wound abscess**	**Operative time (min)**	**Fetal loss**	**Pre-term delivery**	**Post-operative uterine contraction**	**Cesarean section**
Winter et al. (12)	3.7 ± 2 vs. 4.5 ± 2.8	N/A	N/A	N/A	7/125 vs. 0/93	8/125 vs. 8/93	N/A	N/A
Segev et al. (40)	3 ± 2 vs. 5 ± 3	0/50 vs. 5/42	0/50 vs. 0/42	57 ± 26 vs. 60 ± 32	2/50 vs. 2/42	5/50 vs. 3/42	4/50 vs. 5/42	N/A
Laustsen et al. (36)	N/A	1/19 vs. 6/25	0/19 vs. 2/25	N/A	0/19 vs. 0/25	3/19 vs. 2/25	N/A	N/A
McGory et al. (13)	N/A	N/A	N/A	N/A	31/454 vs. 88/2679	1/454 vs. 216/2679	N/A	N/A
Affleck et al. (27)	N/A	N/A	N/A	N/A	0/22 vs. 0/18	3/22 vs. 2/18	N/A	N/A
Carver et al. (29)	2.6 ± 1.6 vs. 2.4 ± 1.4	0/17 vs. 0/11	N/A	N/A	0/17 vs. 0/11	0/17 vs. 0/11	0/17 vs. 1/11	2/17 vs. 3/11
Karaman et al. (34)	3.25 ± 2.45 vs. 4.28 ± 3.31	0/12 vs. 1/36	0/12 vs. 1/36	49.42 ± 11.38 vs. 38.61 ± 11.5	1/12 vs. 1/36	3/12 vs. 9/36	N/A	4/12 vs. 11/36
Cheng et al. (30)	4.4 ± 2.33 vs. 4 ± 1.6	N/A	N/A	N/A	7/128 vs. 37/653	7/128 vs. 74/653	N/A	52/128 vs. 258/653
Peled et al. (38)	3.7 ± 1.1 vs. 3.8 ± 1.3	N/A	N/A	N/A	1/26 vs. 0/59	5/26 vs. 14/59	N/A	7/26 vs. 18/59
Chung et al. (31)	4.2 ± 2.9 vs. 6.9 ± 3.7	0/22 vs. 1/39	1/22 vs. 1/39	44.2 ± 16.4 vs. 47.3 ± 14.7	0/22 vs. 0/39	2/22 vs. 4/39	N/A	6/22 vs. 14/39
Eom et al. (33)	4.5 ± 1.19 vs. 5 ± 3.5	0/15 vs. 0/28	0/15 vs. 1/28	35 ± 16.46 vs. 55 ± 13.75	N/A	0/15 vs. 3/28	1/15 vs. 3/28	N/A
Sadot et al. (39)	3.44 ± 5.4 vs. 4.2 ± 2.1	1/48 vs. 0/17	0/48 vs. 1/17	54 ± 34 vs. 55 ± 25	1/41 vs. 0/16	12/41 vs. 3/16	1/48 vs. 1/17	10/40 vs. 3/16
Cox et al. (32)	2.7 ± 0.7 vs. 3.7 ± 0.7	6/894 vs. 17/441	0/894 vs. 0/441	51.7 ± 2.8 vs. 57.3 ± 3	N/A	0/894 vs. 0/441	N/A	N/A
Gok et al. (18)	1.5 ± 0.5 vs. 1 ± 1.5	1/18 vs. 1/39	0/18 vs. 2/39	48 ± 18 vs. 57 ± 21.25	0/18 vs. 1/39	0/18 vs. 2/39	N/A	N/A
Kirshtein et al. (35)	2.4 ± 1.7 vs. 1.4 ± 0.5	N/A	N/A	29.9 ± 6.3 vs. 28.9 ± 9.2	1/23 vs. 1/19	0/23 vs. 0/19	6/23 vs. 3/19	6/23 vs. 3/19
Lyass et al. (37)	3.55 ± 0.87 vs. 6.1 ± 2.37	0/11 vs. 0/11	0/11 vs. 0/11	61.25 ± 21.66 vs. 46.75 ± 15.88	0/11 vs. 0/11	0/11 vs. 0/11	1/11 vs. 1/11	N/A
Yoo et al. (41)	5.1 ± 2.1 vs. 8.1 ± 10.4	3/24 vs. 2/56	1/24 vs. 4/56	52.8 ± 20.8 vs. 53.9 ± 19.2	3/24 vs. 4/56	2/24 vs. 4/56	N/A	13/24 vs. 18/56
Tumati et al. (19)	2 ± 2 vs. 3 ± 2	N/A	1/547 vs. 0/459	N/A	3/547 vs. 0/459	54/547 vs. 42/459	N/A	231/547 vs. 181/459
Cai et al. (28)	2.83 ± 0.93 vs. 3.78 ± 2.75	0/12 vs. 3/36	1/12 vs. 1/36	71.25 ± 36.29 vs. 80 ± 32.5	1/12 vs. 1/36	1/12 vs. 1/36	N/A	5/12 vs. 20/36
Kapan et al. (26)	1.1 ± 0.32 vs. 1.1 ± 0.32	N/A	N/A	59.5 ± 29.39 vs. 49.2 ± 29.7	0/10 vs. 0/10	N/A	N/A	N/A

*LA, laparoscopic appendectomy; OA, open appendectomy; N/A, not available*.

**Table 3:** Summary outcomes of the meta-analyses.

**Outcomes of interest**	**Studies, *n***	**LA total events**	**LA total patients**	**OA total events**	**OA total patients**	**WMD/OR (95%CI)**	***P-*** **value**	Evidence quality^*^	**Heterogeneity**			
									***X*** **^2^**	**df**	***I*** **^2^,%**	***P*** **-value**
Fetal loss	18	60	1,561	135	4,301	1.93 (1.39, 2.69)	*P* < 0.0001	Moderate	7.53	12	0	*P* = 0.82
Pre-term delivery	19	106	2,460	387	4,760	0.8 (0.48, 1.34)	*P* = 0.40	Low	28.7	14	51	*P* = 0.01
Cesarean section	10	336	851	529	1,384	1.10 (0.91, 1.33)	*P* = 0.34	Very Low	6.55	9	0	*P* = 0.68
Length of stay	17	2.76	1,982	3.84	2,049	−0.57 (−0.96, −0.18)	*P* = 0.004	Low	161.57	16	90	*P* < 0.00001
Operative time	12	51.53	1,137	55.78	774	−2.03 (−6.57, 2.51)	*P* = 0.38	Moderate	43.63	11	75	*P* < 0.00001
Wound infection	12	12	1,142	36	781	0.34 (0.18, 0.62)	*P* = 0.0005	Moderate	12.92	8	38	*P* = 0.11
Wound abscess	12	4	1,672	13	1,229	0.70 (0.28, 1.73)	*P* = 0.43	Low	3.99	8	0	*P* = 0.86
Post-operative uterine contraction	6	13	164	14	128	0.79 (0.36, 1.77)	*P* = 0.57	Very Low	2.39	5	0	*P* = 0.79

Evidence Quality^*^: conducted by the latest GRADEpro software.

*LA, laparoscopic appendectomy; OA, open appendectomy; WMD, weighted mean difference; OR, odds ratio; CI, confidence interval*.

**Table 4:** The assessment of the risk of bias with the Newcastle Ottawa Scale.

**References**	**Selection**				**Comparability (cases and controls)**	**Exposure**			**Scores**
	**Adequate definition of case**	**Representativeness of the cases**	**Selection of controls**	**Definition of controls**		**Ascertainment of exposure**	**Same method of ascertainment for cases and controls**	**Non-response rate**	
Winter et al. (12)	*	*	*	*	**	*	*		8
Segev et al. (40)	*	*	*	*	**	*	*		8
Laustsen et al. (36)	*	*		*	**	*	*		7
McGory et al. (13)	*	*		*	**	*	*	*	8
Affleck et al. (27)	*	*		*	*	*	*		6
Carver et al. (29)	*	*		*	*	*	*	*	7
Karaman et al. (34)	*	*		*	**	*	*	*	8
Cheng et al. (30)	*	*		*	*	*	*	*	7
Peled et al. (38)	*	*		*	*	*	*	*	7
Chung et al. (31)	*	*		*	*	*	*	*	7
Eom et al. (33)	*	*		*	*	*	*	*	7
Sadot et al. (39)	*	*		*	*	*	*	*	7
Cox et al. (32)	*	*		*	*	*	*	*	7
Gok et al. (18)	*	*		*	*	*	*	*	7
Kirshtein et al. (35)	*	*		*	*	*	*		6
Lyass et al. (37)	*	*		*	**	*	*	*	8
Yoo et al. (41)	*	*		*	*	*	*	*	7
Tumati et al. (19)	*	*		*	**	*	*	*	8
Cai et al. (28)	*	*		*	**	*	*	*	8
Kapan et al. (26)	*	*		*	*	*	*	*	7

* *Represents one point.*

** *Represents two points*.

### Meta-Analysis Outcomes

#### Fetal Loss

**Figure 2:**
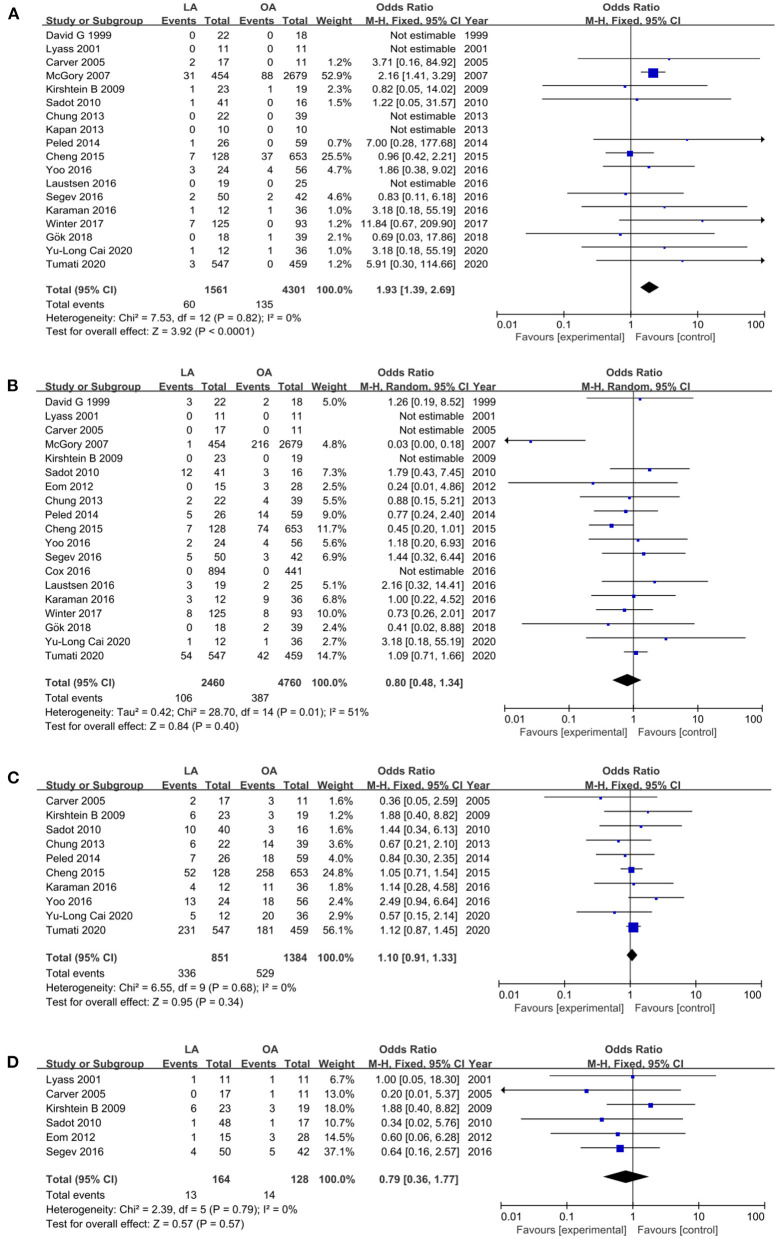
Forest plot of the obstetrical outcomes comparison of LA vs. OA. **(A)** Forest plot for fetal loss (OR 1.93, 95% CI 1.39–2.69, *P* < 0.0001); **(B)** Forest plot for pre-term delivery (OR 0.80, 95% CI 0.48–1.34, *P* = 0.40); **(C)** Forest plot for cesarean section (OR 1.10, 95% CI 0.91–1.33, *P* = 0.34); **(D)** Forest plot for post-operative uterine contraction (OR 0.79, 95% CI 0.36–1.77, *p* = 0.57).

Eighteen studies (12, 13, 18, 19, 26–31, 34–41) with a total of 5,862 patients (1,561 who underwent LA and 4,301 who underwent OA) recorded fetal loss. The pooled results suggested that there were 60 (3.8%) fetal losses in the LA in contrast to 135 (3.1%) in the OA from eighteen trials (OR 1.93, 95% CI 1.39–2.69, *P* < 0.0001). Heterogeneity analysis showed no statistically significant difference (*I*^2^ = 0%); therefore, a fixed effects model (FEM) was applied (Figure 2A).

#### Pre-term Delivery

Pre-term delivery, defined as labor before 37 gestational weeks, was assessed in 19 studies (12, 13, 18, 19, 27–41). The results suggested that there was no difference in pre-term delivery in the two groups (OR 0.80, 95% CI 0.48–1.34, *P* = 0.40). Heterogeneity was medium (*I*^2^ = 51%) and analyzed in a random effects model (REM) (Figure 2B).

#### Cesarean Section

Ten studies (19, 28–31, 34, 35, 38, 39, 41) that encompassed 2,235 patients (851 in LA and 1,384 in OA) recorded the cesarean section, and the pooled data revealed no statistical difference in cesarean section (OR 1.10, 95% CI 0.91–1.33, *P* = 0.34). There was no statistically significant heterogeneity (*I*^2^ = 0%) and was analyzed in FEM (Figure 2C).

#### Post-operative Uterine Contraction

Six trials (29, 33, 35, 37, 39, 40) reported post-operative uterine contraction, and the pooled results suggested no statistically significant difference in post-operative uterine contraction (OR 0.79, 95% CI 0.36–1.77, *p* = 0.57, *I*^2^ = 0%, Figure 2D).

#### Operative Time

**Figure 3:**
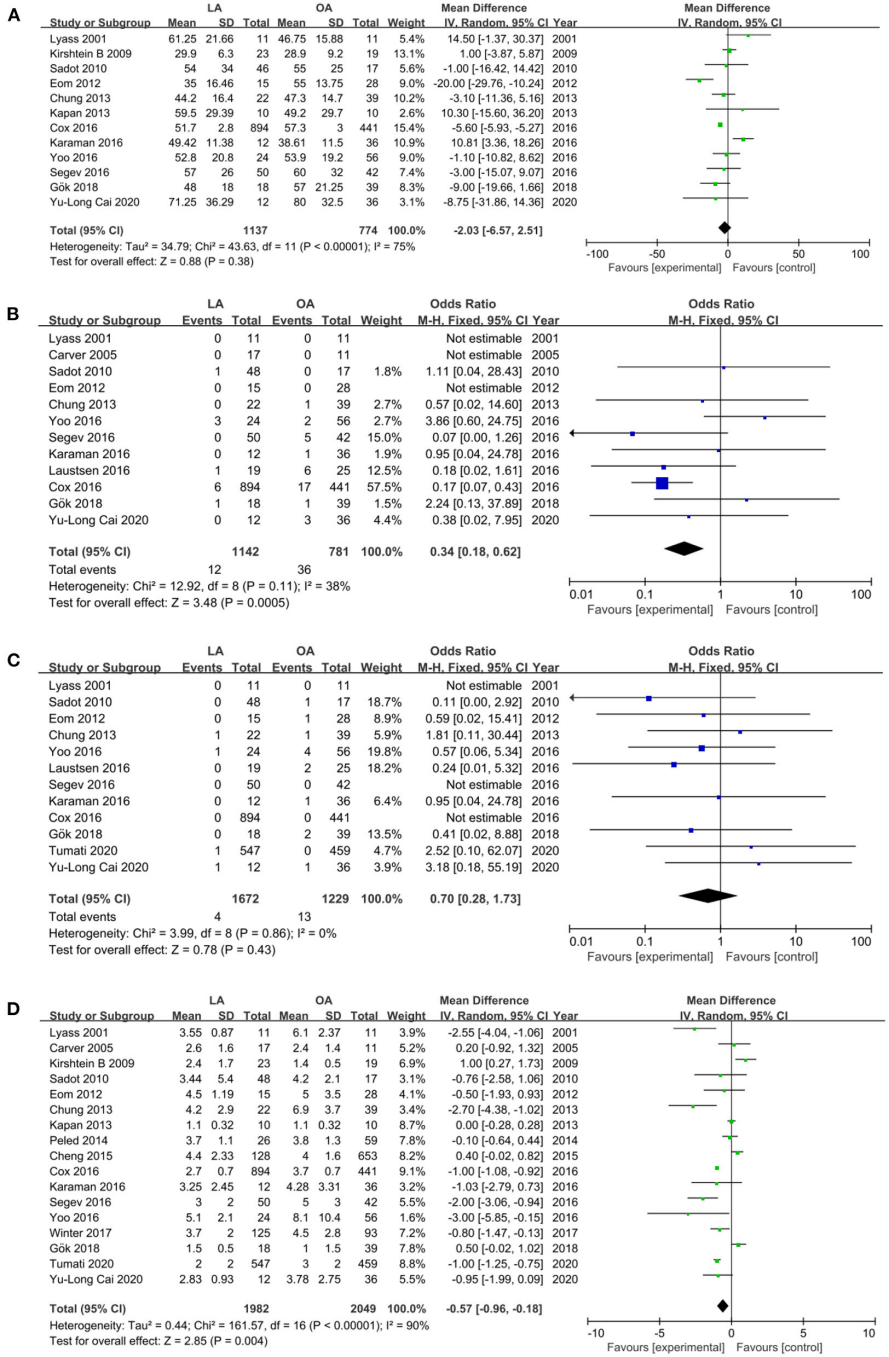
Forest plot of the surgical outcomes comparison of LA vs. OA. **(A)** Forest plot for operative time (MD −2.03 min, 95% CI −6.57 to 2.51, *P* = 0.38); **(B)** Forest plot for wound infection (OR 0.34, 95% CI 0.18– 0.62, *P* = 0.0005); **(C)** Forest plot for abscess (OR 0.70, 95% CI 0.28–1.73, *p* = 0.43); **(D)** Forest plot for LOS (MD −0.57 days, 95% CI −0.96 to −0.18, *P* = 0.004).

Estimated operative time was assessed in 12 studies (18, 26, 28, 31–35, 37, 39–41). The pooled results showed that there was no statistical difference in operative time (MD −2.03 min, 95% CI −6.57 to 2.51, *P* = 0.38). Heterogeneity was high and was analyzed in the REM (*I*^2^ = 75%) (Figure 3A).

#### Wound Infection and Abscess

Wound infection (18, 28, 29, 31–34, 36, 37, 39–41) and abscess (18, 19, 28, 31–34, 36, 37, 39–41), two crucial perioperative outcomes, were reported in diverse 12 studies. Patients undergoing LA had lower wound infection compared with OA (OR 0.34, 95% CI 0.18–0.62, *P* = 0.0005, *I*^2^ = 38%, Figure 3B), whereas, there was no statistical difference in wound abscess (OR 0.70, 95% CI 0.28–1.73, *p* = 0.43, *I*^2^ = 0%, Figure 3C).

#### Length of Stay

The LOS was referred in 17 studies (12, 18, 19, 26, 28–35, 37–41) with a total of 4,031 patients (1,982 and 2,049 underwent LA and OA, respectively). The results revealed a shorter LOS in the LA (MD −0.57 days, 95% CI −0.96 to −0.18, *P* = 0.004) (Figure 3D). Heterogeneity was high (*I*^2^ = 90%) and analyzed in REM.

#### Sensitivity Analysis and Quality Assessment

The remaining parameters were not impacted after each successive exclusion of the eligible literature. All included publications had NOS scores ≥ 6, indicating a low risk of bias.

#### Publication Bias

**Figure 4:**
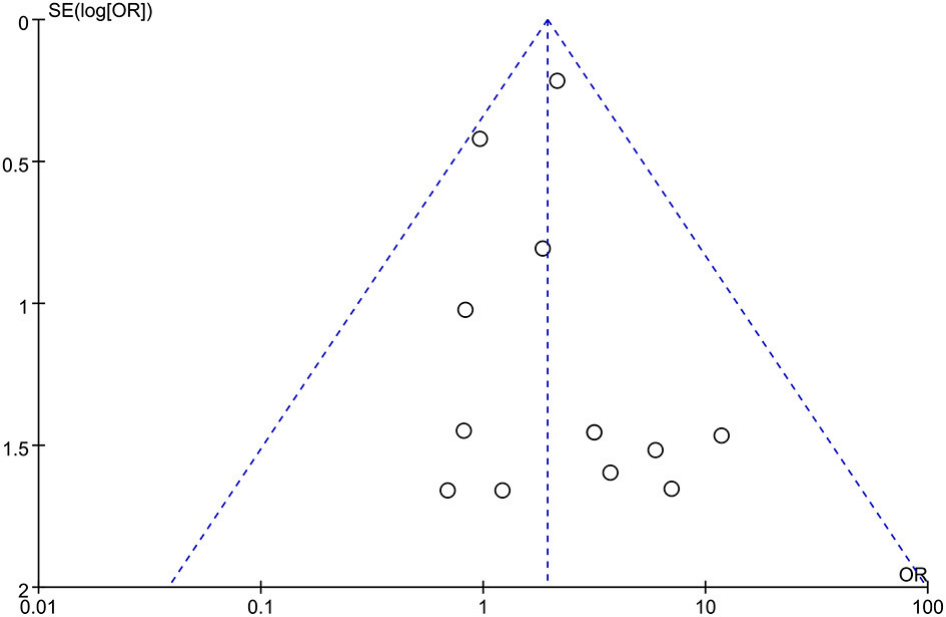
Funnel plot for Fetal loss.

Begg funnel plots were depicted for each result to estimate publication bias. All studies were within 95% CIs in the funnel plot of fetal loss (Figure 4), which suggested no obvious publication bias.

## Discussion

Nowadays, there is deficient testimony to point out the optimal operation of appendicitis in pregnancy because large RCTs are difficult to conduct in pregnant women. In recent years, systematic reviews and meta-analyses also had no consensus on whether to choose LA or OA (15–17). Under such an unclear situation, further explorations for pregnant appendicitis are necessary.

This systematic review summarized the knowledge and conclusions of the current literature. In our results, LA for pregnancy appendicitis had advantages in the aspect of hospital stay and wound infection. Nevertheless, operative time and the general post-operative complications, wound abscess and post-operative uterine contractions, did not differ among LA and OA. As for obstetric outcomes, the laparoscopic operation was two times as likely to cause fetal loss as compared with OA; while pre-term delivery and cesarean section were comparable between the two operations.

The average hospital stay in the laparoscopic group was reduced by half a day, to some extent. Shorter hospital stay meant cost savings and improved patient satisfaction (42). LA had higher expenses in the terms of material cost than open appendicectomy. However, some studies had shown no obvious diversity in total hospitalization costs between the two approaches (28), reduction in hospital stay for LA might have some contribution. Compared with traditional open appendectomy, LA was a relatively rising operation, but the results of our analysis revealed that surgery time between the two operation methods was almost similar. It potentially showed the increase in experience and learning curve of laparoscopic appendectomy over the last 20 years. Laparoscopic operations had predominance in some post-operative complications such as wound infection, according to the previous experience and literatures (10, 43). This is one reason that laparoscopy is more, in general, used for treating pregnant acute appendicitis.

**Figure 5:**
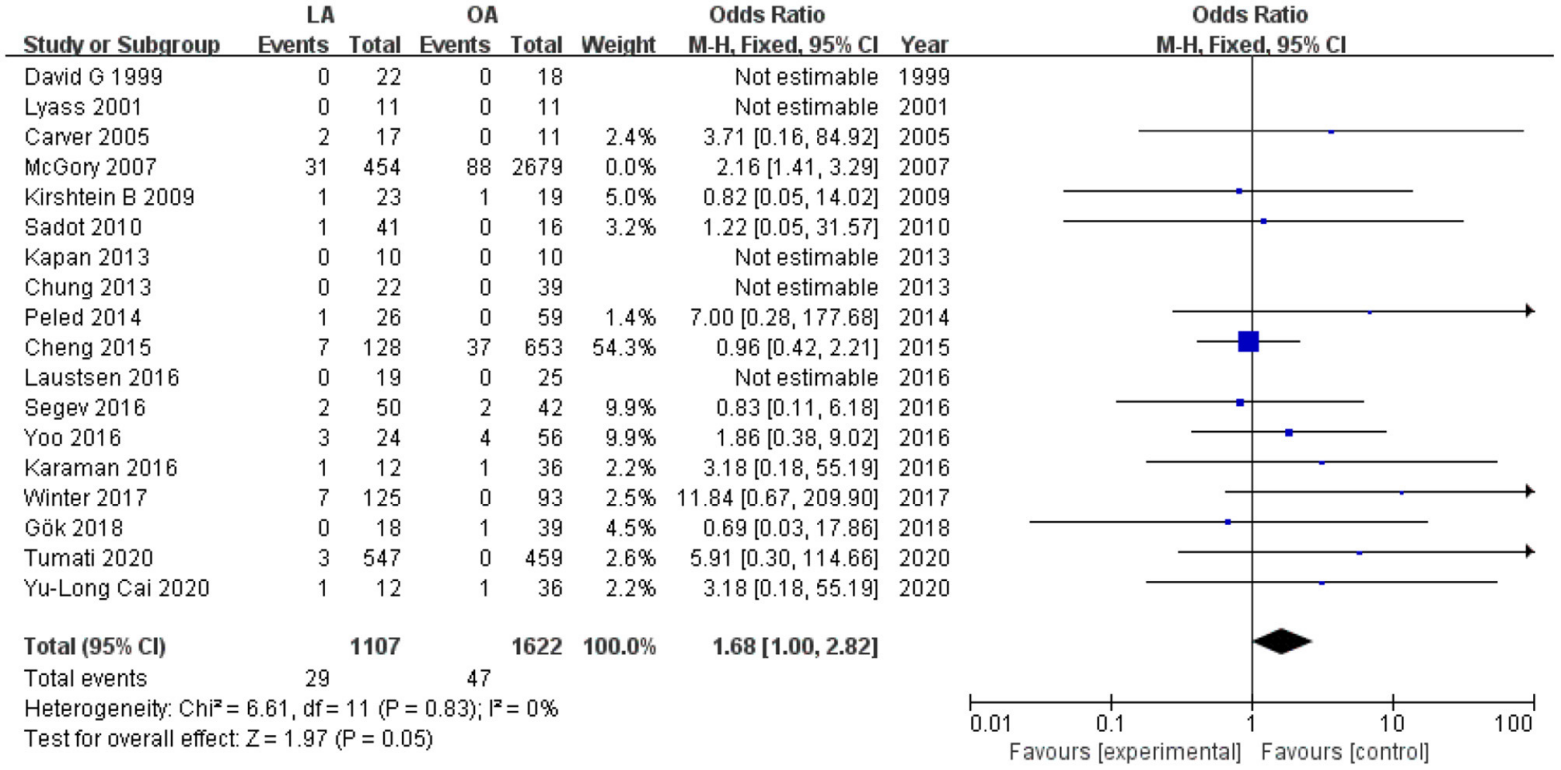
Forest plot for Fetal loss after excluding the study by McGory et al.

Fetal loss is the most devastating and frightening complication of surgical intervention for pregnancy appendicitis. In our conclusion, fetal loss in the laparoscopic group was significantly higher than OA. The pooled risk was 1.93, similar to the meta-analysis and systematic review carried out by Wilasrusmee et al. (43), in which the risk of fetal loss in LA was 1.91 times that in OA. Our study and the study by Wilasrusmee both included a large sample size study conducted by McGory et al. (13). The patient-volume of this study accounted for about 40% of our analysis. When excluded it from the included studies, Wilasrusmee et al. considered that there was no association between surgical methods and fetal loss. However, our results showed that the risk of fetal loss in LA was 1.68 times higher than that in OA after the above-mentioned large sample study was removed (*p* = 0.05, Figure 5). Although *p* = 0.05, the OR value still reminded us of a trend that laparoscopic appendectomy was linked with a higher risk and possibility of fetal loss. With the progress from simple to complicated appendicitis, which meant the increase of infection, the risk of fetal death and maternal morbidity showed an increasing trend (6). Complicated appendicitis was defined as suppurative, perforated, gangrenous appendicitis, or combined peritonitis appendicitis. We analyzed the distribution of complicated appendicitis between LA and OA groups (OR 0.69, 95% CI 0.59–0.81, *p* < 0.00001, Supplementary Figure 1), suggesting that complicated appendicitis in the LA group was significantly less than in the OA group. In the case of mild appendicitis infection, there was still a higher rate of fetal loss for laparoscopic appendectomy, highlighting the correlation between fetal loss and laparoscopy itself.

Therefore, it is quite necessary for patients and doctors to fully evaluate the risk of fetal loss associated with LA. Perioperative and operational management should be enhanced and executed by a multidisciplinary team. In addition to skilled and prudent operation, the signing of informed consent is also very significant. The increase of fetal loss during LA may be closely related to ascending intraperitoneal pressure and fetal carbon dioxide acidosis caused by CO_2_ pneumoperitoneum (44). The potential harm to the pregnant uterus during trocar passage and operation also possibly led to abortion (37, 45). Increasing abdominal pressure along with Trendelenburg's position also had the potential risk of affecting the infant by leading to maternal hypercapnia and hypoxemia (46). Pneumoperitoneum status for individual study, including the magnitudes of intraabdominal pressure and the types of pneumoperitoneum, are presented in Supplementary Table 1. Almost all included studies mentioned that laparoscopic appendectomy was performed in pneumoperitoneum circumstances. Ten studies explicitly stated that the pneumoperitoneum was produced by CO_2_, and nine of them showed the specific range of intraabdominal pressure, all of which ranged from 10 to 15 mmHg. Recent SAGES guidelines inclined to 10–15 mmHg CO_2_ injection as a relatively safe procedure for laparoscopy in pregnant patients (8). Controlling the pressure and gas may be a feasible way to reduce the probability of fetal loss; however, large-scale and well-designed studies were required to demonstrate this perspective.

Post-operative uterine contraction, correlated with pre-term delivery, is one of the common complications after appendicectomy for pregnant women. In our results, pre-term delivery and post-operative uterine contraction were both fairly comparable among the two operations, which might illustrate that there was no noteworthy correlation between surgical methods and these two indicators.

Calculating the overall rate of pre-term delivery in all 20 studies (*R* = 54 + 42/547 + 459 ^*^ 100% = 9.5%), we found it was almost similar to the global incidence of pre-term birth (47). Spontaneous abortion accounted for 10–20% of all pregnancies worldwide. However, the fetal loss among all pregnancy appendicitis in our systematic review was much lower, at about 3.3%. This might be because the fetal loss in this study was only related to surgical and infectious factors, whereas spontaneous abortion in the world was related to various factors such as genes, endocrine, immunity, and environment.

To avoid data loss and the influence of unnecessary confounding factors, two authors independently searched the literature. Meanwhile, setting the criteria that study < 10 patients per group and studies that appendectomy was mixed with other operations and could not be differentiated were excluded. Even so, there were many inherent limitations in the present analysis. First, all the 20 including trials were retrospective studies, implying inevitable follow-up bias and selective bias. Second, the time interval of the included literature was quite long (20 years), and the management philosophy of pregnant appendicitis patients evolved over time. In addition, the comparability between our present research and the anterior study by Wilasrumsee et al. seemed to be another limitation. Third, for third-trimester patients, doctors and patients were more inclined to choose open appendectomy, as the insertion and operation of laparoscopic devices could cause damage to the enlarged uterus (40). Our review also showed that the gestational age at surgery in the open group was significantly higher than laparoscopy (MD = −3.31, 95% CI −4.27 to −2.35; *P* < 0.00001, Supplementary Figure 2). Therefore, the distribution of trimesters in the two groups potentially generated bias between them. In addition, the weights and pre-existing abdominal incisions of the patient, the choice of surgeons, and their experience were all likely to affect the final procedure. The included studies did not provide outcomes in different trimesters, impeding to get definitive results of the optimal and feasible surgical procedure in the three diverse trimesters (Table 1). Fourth, in the included studies, there were cases where LA was converted to open; about 11 out of the 2,477 patients of LA were transformed to OA. Six, nearly half of the 11 cases, were still classified as LA. Those transformations have possibly taken confounders to the outcomes. When we retrieved databases, we found vast clinical studies about laparoscopic appendectomy for pregnant appendicitis, partly reflecting that LA has widely been used in clinical practice. The applicability and feasibility of LA could be naturally interpreted to some degree.

## Conclusion

Laparoscopic appendectomy is safe in experienced hands, with the admitted advantages of minimally invasive surgery, especially in hospital stays and the common post-operative complications. However, the obvious increase in fetal loss compared with OA should never be ignored. All in all, laparoscopic appendectomy should be undertaken by a comprehensive team, and it is essential for patients to be thoroughly informed about the merits and demerits of the available surgical methods. Further, larger-scale and well-designed studies are demanded to elucidate the reasons for the high incidence of fetal loss after laparoscopic appendectomy.

## Data Availability Statement

The original contributions presented in the study are included in the article/Supplementary Material, further inquiries can be directed to the corresponding author.

## Author Contributions

JZ: data collection, data analysis, and writing. QF: study design and modification. PL: data collection. MW: make form. ZX: data collection and make form. All authors contributed to the article and approved the submitted version.

## Funding

Model research and application demonstration of hierarchical coordination within health alliance based on a cloud platform (Project No.: 2020YFS0092).

## Conflict of Interest

The authors declare that the research was conducted in the absence of any commercial or financial relationships that could be construed as a potential conflict of interest.

## Publisher's Note

All claims expressed in this article are solely those of the authors and do not necessarily represent those of their affiliated organizations, or those of the publisher, the editors and the reviewers. Any product that may be evaluated in this article, or claim that may be made by its manufacturer, is not guaranteed or endorsed by the publisher.

## Supplementary Material

Supplementary Figure 1 Forest plot for the distribution of complicated appendicitis between LA and OA groups.

Supplementary Figure 2 Forest plot for the gestational age at the surgery between LA and OA groups.

Supplementary Table 1 Pneumoperitoneum status of included studies.

Appendix The particular search strategy.
