# Supplementary figures and images for: Updated Evaluation of Laparoscopic vs. Open Appendicectomy During Pregnancy: A Systematic Review and Meta-Analysis
Source: Front Surg. 2021 Sep 23;8:720351. doi: 10.3389/fsurg.2021.720351 (PMC8495069; doi:10.3389/fsurg.2021.720351)

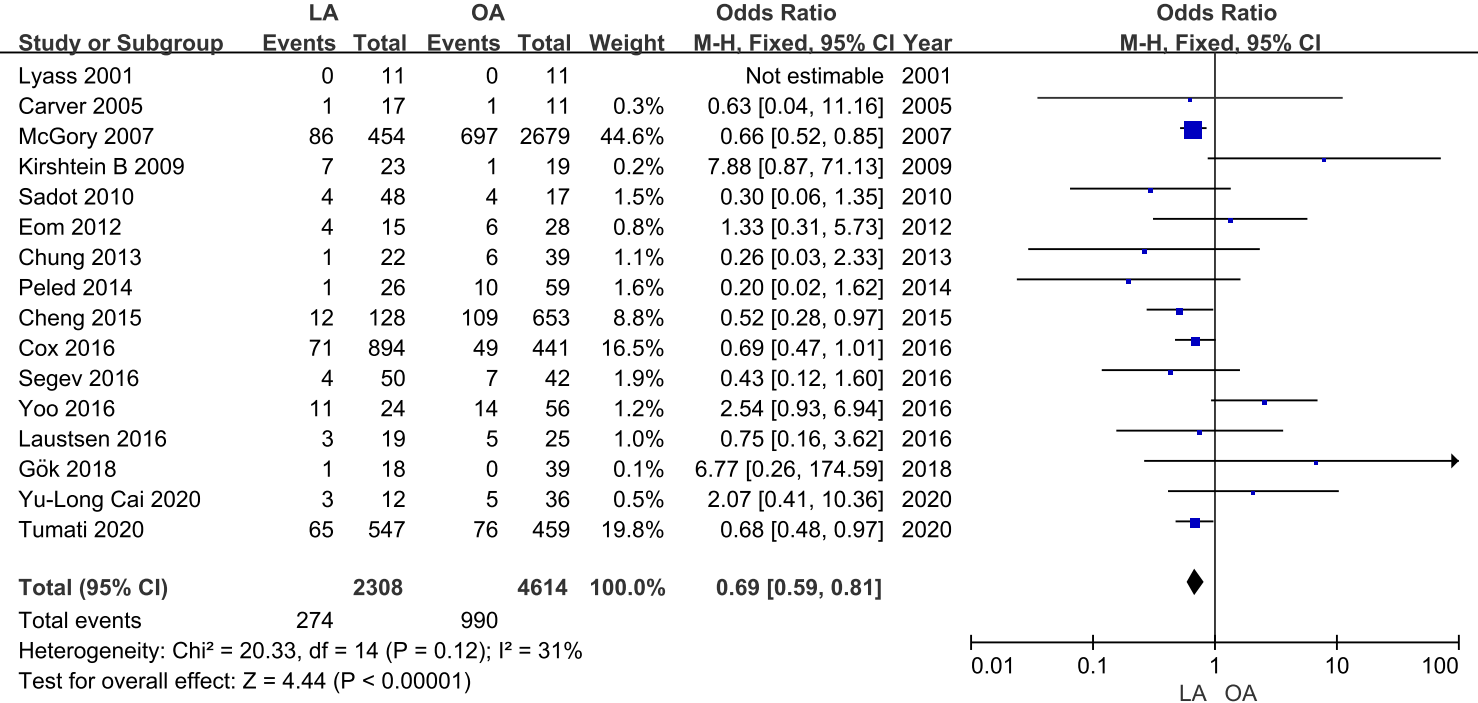

Supplement: Supplementary Figure 1 — Forest plot for the distribution of complicated appendicitis between LA and OA groups. [file Data_Sheet_1.PDF]

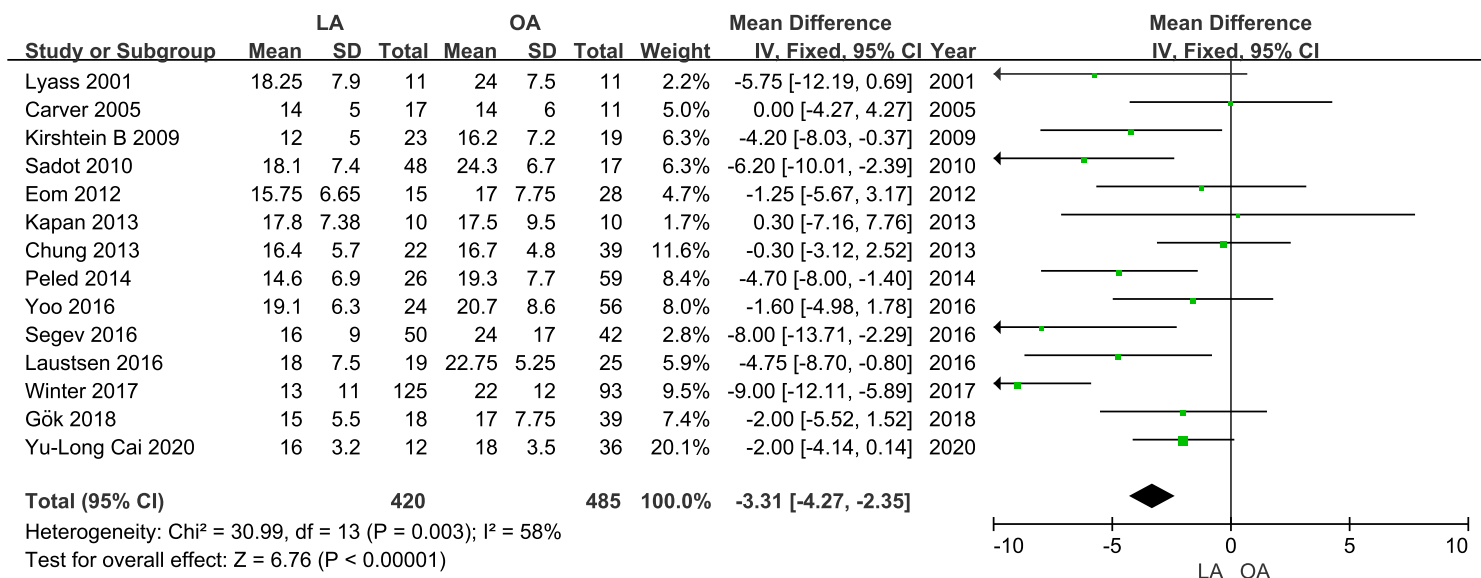

Supplement: Supplementary Figure 2 — Forest plot for the gestational age at the surgery between LA and OA groups. [file Data_Sheet_2.PDF]
