# Supplementary material for: Updated Evaluation of Laparoscopic vs. Open Appendicectomy During Pregnancy: A Systematic Review and Meta-Analysis
Source: Front Surg. 2021 Sep 23;8:720351. doi: 10.3389/fsurg.2021.720351 (PMC8495069; doi:10.3389/fsurg.2021.720351)
Supplement: Appendix — The particular search strategy. [file Data_Sheet_3.PDF]

#1.("Pregnancy"[Mesh]) OR (((((((Pregnancies[Title/Abstract]) OR (Gestation[Title/Abstract])) OR (Gestations[Title/Abstract])) OR (Pregnant Women[Title/Abstract])) OR (Pregnant Woman[Title/Abstract])) OR (Woman, Pregnant[Title/Abstract])) OR (Women, Pregnant[Title/Abstract]))

#2.("Laparoscopy"[Mesh]) OR ((((((((((((((((((((((((Laparoscopies[Title/Abstract]) OR (Laparoscopes[Title/Abstract])) OR (Peritoneoscopes[Title/Abstract])) OR (Peritoneoscopy[Title/Abstract])) OR (Peritoneoscope[Title/Abstract])) OR (Peritoneoscopies[Title/Abstract])) OR (Celioscopes[Title/Abstract])) OR (Celioscopy[Title/Abstract])) OR (Celioscope[Title/Abstract])) OR (Celioscopies[Title/Abstract])) OR (Laparoscope[Title/Abstract])) OR (Surgical Procedures, Laparoscopic[Title/Abstract])) OR (Laparoscopic Surgical Procedure[Title/Abstract])) OR (Procedure, Laparoscopic Surgical[Title/Abstract])) OR (Procedures, Laparoscopic Surgical[Title/Abstract])) OR (Surgery, Laparoscopic[Title/Abstract])) OR (Laparoscopic Surgical Procedures[Title/Abstract])) OR (Laparoscopic Surgery[Title/Abstract])) OR (Laparoscopic Surgeries[Title/Abstract])) OR (Surgeries, Laparoscopic[Title/Abstract])) OR (Laparoscopic Assisted Surgery[Title/Abstract])) OR (Laparoscopic Assisted Surgeries[Title/Abstract])) OR (Surgeries, Laparoscopic Assisted[Title/Abstract])) OR (Surgery, Laparoscopic Assisted[Title/Abstract])) OR (Surgical Procedure, Laparoscopic[Title/Abstract])).

#3. ("Laparotomy"[Mesh]) OR ((((((((((((((((((((((((((Laparotomies[Title/Abstract]) OR (Minilaparotomy[Title/Abstract])) OR (Minilaparotomies[Title/Abstract])) OR (open[Title/Abstract])) OR (Open Surgical Procedure[Title/Abstract])) OR (Open Procedure[Title/Abstract])) OR (open approach[Title/Abstract])) OR (open Surgical approach[Title/Abstract])) OR (conventional Surgical Procedure[Title/Abstract])) OR (conventional Procedure[Title/Abstract])) OR (conventional approach[Title/Abstract])) OR (conventional Surgical approach[Title/Abstract])) OR (Surgical Procedures, open[Title/Abstract])) OR (Procedures, open Surgical[Title/Abstract])) OR (Surgery,

open[Title/Abstract])) OR (open Surgical Procedures[Title/Abstract])) OR (open Surgery[Title/Abstract])) OR (open Surgeries[Title/Abstract])) OR (Surgeries, open[Title/Abstract])) OR (Surgical Procedure, open[Title/Abstract])) OR (Surgical Procedures, conventional[Title/Abstract])) OR (Procedures, conventional Surgical[Title/Abstract])) OR (Surgery, conventional[Title/Abstract])) OR (conventional Surgical Procedures[Title/Abstract])) OR (conventional Surgery[Title/Abstract])) OR (conventional Surgeries[Title/Abstract])) OR (Surgeries, conventional[Title/Abstract])) OR (Surgical Procedure, conventional[Title/Abstract])).

#4. ("Appendectomy"[Mesh]) OR (Appendectomies[Title/Abstract]).

#5. (((Randomized Controlled Trials[MeSH]) OR (Clinical Trials, Randomized)) OR (Trials, Randomized Clinical)) OR (Controlled Clinical Trials, Randomized).

#6.(((Retrospective Studies[MeSH]) OR (Studies, Retrospective)) OR (Study, Retrospective)) OR (Retrospective Study).

#7. (((Case-Control Studies[MeSH]) OR (Case-Control Study)) OR (Studies, Case-Control)) OR (Study, Case-Control)) OR (Case-Comparison Studies)) OR (Case Comparison Studies)) OR (Case-Comparison Study)) OR (Studies, Case-Comparison)) OR (Study, Case-Comparison)) OR (Case-Compeer Studies)) OR (Studies, Case-Compeer)) OR (Case-Referrent Studies)) OR (Case Referrent Studies)) OR (Case-Referrent Study)) OR (Studies, Case-Referrent)) OR (Study, Case-Referrent)) OR (Case-Referent Studies)) OR (Case Referent Studies)) OR (Case-Referent Study)) OR (Studies, Case-Referent)) OR (Study, Case-Referent)) OR (Case-Base Studies)) OR (Case Base Studies)) OR (Studies, Case-Base)) OR (Case Control Studies)) OR (Case Control Study)) OR (Studies, Case Control)) OR (Study, Case Control)) OR (Nested Case-Control Studies)) OR (Case-Control Studies, Nested)) OR (Case-Control Study, Nested)) OR (Nested Case Control Studies)) OR (Nested Case-Control Study)) OR (Studies, Nested Case-Control)) OR (Study, Nested Case-Control)) OR (Matched Case-

Control Studies)) OR (Case-Control Studies, Matched)) OR (Case-Control Study, Matched)) OR (Matched Case Control Studies)) OR (Matched Case-Control Study)) OR (Studies, Matched Case-Control)) OR (Study, Matched Case-Control).

#8. (((((((((((((((((((((((((((Cohort Studies[MeSH]) OR (Cohort Study)) OR (Studies, Cohort)) OR (Study, Cohort)) OR (Concurrent Studies)) OR (Studies, Concurrent)) OR (Concurrent Study)) OR (Study, Concurrent)) OR (Closed Cohort Studies)) OR (Cohort Studies, Closed)) OR (Closed Cohort Study)) OR (Cohort Study, Closed)) OR (Study, Closed Cohort)) OR (Studies, Closed Cohort)) OR (Analysis, Cohort)) OR (Cohort Analysis)) OR (Analyses, Cohort)) OR (Cohort Analyses)) OR (Historical Cohort Studies)) OR (Cohort Study, Historical)) OR (Historical Cohort Study)) OR (Study, Historical Cohort)) OR (Cohort Studies, Historical)) OR (Studies, Historical Cohort)) OR (Incidence Studies)) OR (Incidence Study)) OR (Studies, Incidence)) OR (Study, Incidence).
